# Effectiveness and safety of periareolar micro-excision combined with liposuction for gynecomastia: a retrospective cohort of 177 patients

**DOI:** 10.3389/fsurg.2026.1822281

**Published:** 2026-06-18

**Authors:** Xinxin Guo, Baoji Song

**Affiliations:** Department of Plastic Surgery, Tianjin Hospital, Tianjin, China

**Keywords:** fat, gynecomastia, liposuction, periareolar micro-incision, plastic surgery

## Abstract

**Objective:**

Gynecomastia is common and carries substantial psychosocial burden; liposuction alone has limited efficacy for glandular removal. This study evaluated the effectiveness and safety of liposuction combined with periareolar micro-incision gland excision for the treatment of gynecomastia.

**Methods:**

From January 2019 to April 2025, we treated 177 patients with gynecomastia. All underwent liposuction plus periareolar micro-incision gland excision. We performed a retrospective review of clinical records to assess long-term surgical outcomes, complications, and patient satisfaction.

**Results:**

The median excised gland weight was 41.9 g (range, 8–180 g); the median lipoaspirate volume was 882 mL (range, 100–2,500 mL). The median operative time was 89.7 min (range, 60–120 min). Complications included seroma in 15 patients, hematoma in 3 patients within 6 h postoperatively, infection in 3 patients, and transient cutaneous sensory changes in 4 patients. The mean patient-reported satisfaction score was 8.8/10.

**Conclusions:**

Liposuction combined with periareolar micro-incision gland excision is a technically feasible and cosmetically favorable treatment for gynecomastia. Although postoperative wrinkling or folding of the nipple–areolar complex may occur in patients with more severe deformities, the technique can still provide satisfactory aesthetic outcomes while keeping visible scarring to a minimum. Attention to uniform tumescent infiltration, meticulous hemostasis, dead-space control, and staged compression may further reduce early hematoma and seroma and help prevent postoperative asymmetry, particularly in patients with higher BMI.

## 1 Introduction

Gynecomastia is an enlargement of the male breast caused by benign proliferation of glandular tissue. Most cases are idiopathic with a self-limiting course. The condition is relatively common, with reported prevalence ranging from 32% to 72% (1, 2). Beyond physical signs, gynecomastia imposes a substantial psychosocial burden, commonly manifesting as depression, anxiety, impaired self-esteem, gender/identity-related distress, and social avoidance.

Clinically, many patients are asymptomatic, though mastalgia or pruritus may occur. Earlier initiation of pharmacotherapy after symptom onset is associated with better outcomes, whereas persistent symptoms for ≥1 year and poor response to medication favor surgical intervention (1). Contemporary surgical options include liposuction, subcutaneous mastectomy, skin-sparing mastectomy, mastectomy with skin excision, and mastectomy with free nipple grafting (3). Advances in liposuction provide a less invasive route that can markedly improve chest contour by removing excess fat (4), but its ability to eliminate glandular tissue is limited. Consequently, periareolar minimally invasive gland excision combined with liposuction has become a mainstream approach, enabling more thorough gland removal while preserving contour sculpting to enhance efficacy and reduce recurrence risk (5, 6). In parallel, endoscope-assisted techniques offer superior visualization and instrumentation for precise dissection and excision (7, 8).

Against this background, the present study aims to systematically evaluate the efficacy and safety of periareolar minimally invasive gland excision plus liposuction for gynecomastia. We performed longitudinal pre- and postoperative assessments and focused on key outcome measures, including aesthetic/contour improvement, postoperative complication rates, and patient-reported satisfaction.

Through multi–time-point follow-up and continuous monitoring of complications and satisfaction, we seek to delineate the benefits and limitations of this combined approach in terms of therapeutic gain and risk control, thereby providing robust evidence to inform individualized surgical decision-making and procedural optimization.

## 2 Methods

**Figure 1:**
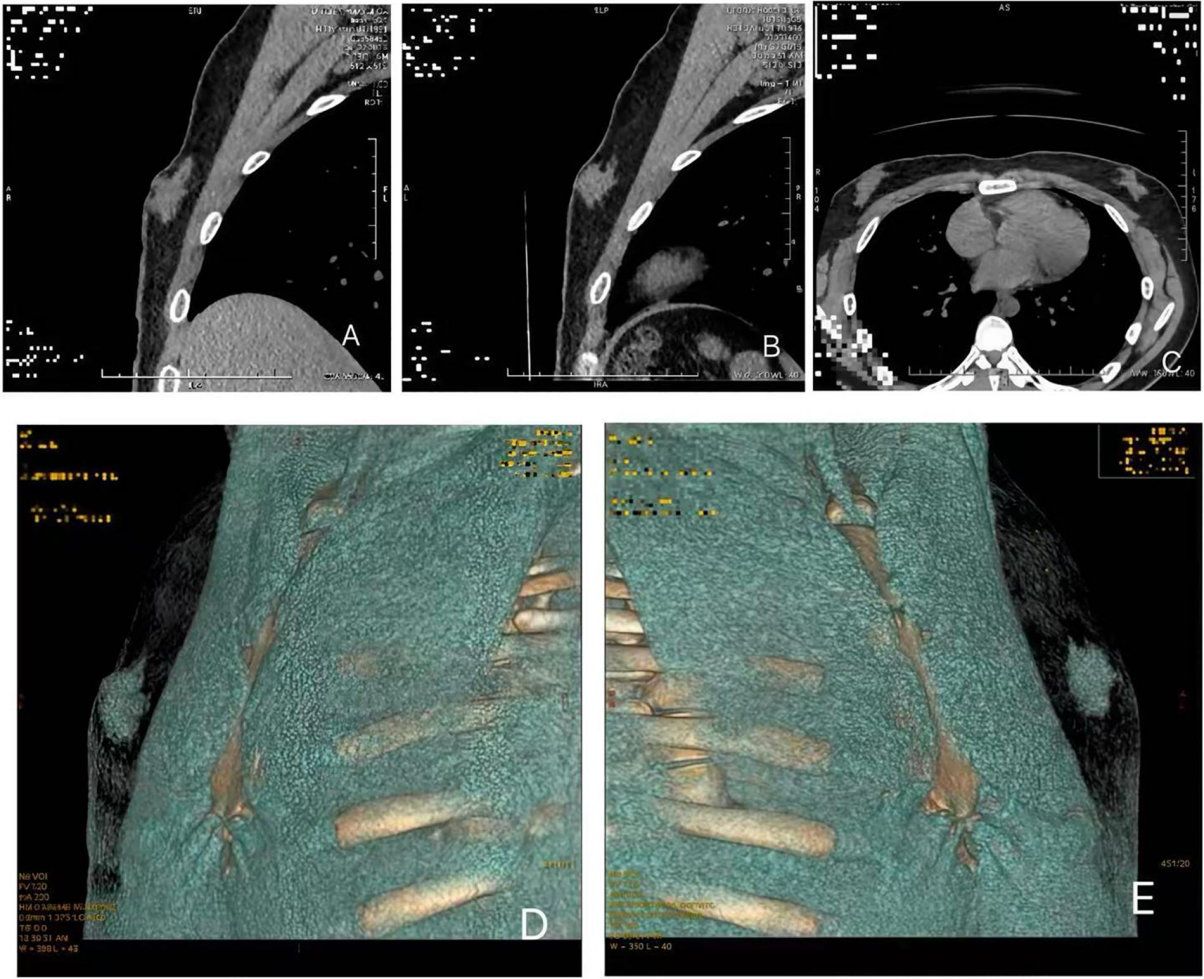
A 34-year-old male patient shown preoperative low-dose CT images at **(A)** left sagittal, **(B)** right sagittal and **(C)** horizontal view; and preoperative at **(D)** left 3D reconstruction and **(E)** right 3D reconstruction views.

We conducted a single-center, single-surgeon retrospective cohort of consecutive patients who underwent surgical treatment for gynecomastia at our institution between January 2019 and April 2025. Preoperative assessment included a detailed medical history and physical examination, 3D chest CT, routine hematologic and biochemical testing, and a standardized sex-hormone panel. Intraoperative reports and perioperative clinical records were reviewed by the study team, and disease severity was classified using the Rohrich grading system. In total, 177 patients were enrolled; all patients underwent preoperative low-dose three-dimensional breast CT imaging (Figure 1), all procedures were performed by the same surgeon to minimize operator-related confounding. Written informed consent was obtained from all participants. Clinical photographs were acquired under standardized conditions preoperatively and at scheduled follow-ups—or, when applicable, submitted electronically by patients—with explicit authorization for research use and academic publication. In the absence of a widely validated, gynecomastia-specific outcome instrument, we developed and implemented a center-specific patient-satisfaction questionnaire (Table 1) to quantitatively assess postoperative outcomes.

**Table 1:** Patient satisfaction questionnaire (G-PRO-10).

Evaluation items	Dissatisfied	Neutral	Satisfied
Overall chest appearance			
Masculine chest contour (flatness and natural definition)			
Left–right chest symmetry			
Contour smoothness (no obvious depression, bulging, or surface irregularity)			
Nipple–areolar complex shape and position			
Recovery of nipple–areolar sensation			
Scar visibility (axillary and periareolar scars)			
Postoperative pain and recovery course			
Preoperative communication and postoperative follow-up experience			
Improved confidence and social comfort after surgery			

Each item is rated on a 3-point scale (0 = dissatisfied, 0.5 = neutral, 1 = very satisfied). The total raw score is the sum of all items, with higher scores indicating greater satisfaction.

### 2.1 Preoperative assessment and marking

The patient is positioned standing for preoperative surface marking, with standardized photographic documentation. Key anthropometric distances and symmetry parameters are measured and recorded using defined anatomical landmarks. Based on the surface assessment, planned liposuction zones are delineated around the breast and along the lateral chest wall. Beyond removing abnormal glandular and adipose tissue, the surgical plan prioritizes precise contour refinement and skin redraping to achieve a natural, masculine chest appearance.

### 2.2 Surgical technique

All procedures were performed under intravenous anesthesia. Patients were positioned supine with both upper limbs abducted to fully expose the anterior chest and axillae. A single 4-mm stab incision was placed along the axillary crease at the apex of the mid-axillary line, within the hair-bearing area for optimal concealment. Through this access, a tumescent solution (1 L 0.9% saline + 20 mL lidocaine 2% + 10 mL ropivacaine 0.5% + 1 mL epinephrine + 1 g tranexamic acid) was infiltrated into the gland and planned liposuction zones; a dwell time of approximately 10 min was observed to ensure uniform tissue perfusion, minimize bleeding, optimize analgesia, and preserve a natural postoperative chest contour.

Liposuction was then performed with a 4-mm blunt-tip cannula in the superficial-to-mid subcutaneous plane over the periareolar and lateral chest to improve the thoracic–anterior chest transition and promote skin remodeling. Particular attention was paid to the fibro-adipose bands surrounding the gland to facilitate smooth contour transitions. Endpoints were determined by the desired chest shape and bilateral symmetry, avoiding over-suction that could lead to dimpling or adhesions.

Gland excision was carried out via 0.5 cm periareolar micro-incisions at 5–6 o'clock on the right areola and 6–7 o'clock on the left. Sharp dissection between the gland and the subdermal plane was performed using a curved, fine toothed Metzenbaum scissors (≈12 cm), followed by traction-assisted excision with fine-toothed forceps to achieve complete removal of hyperplastic tissue. The pectoralis major fascia was preserved to minimize adhesions and contour irregularities. To protect the vascularity of the nipple–areolar complex (NAC), approximately 1 cm of subareolar glandular tissue was retained; the contralateral side was treated identically.

After meticulous hemostasis, tension-reducing layered closure was performed with 4-0 absorbable sutures, and a sterile skin adhesive was applied to the periareolar micro-incisions as needed. If necessary, limited finishing liposuction was performed through the original axillary incision to achieve final contour refinement.

**Figure 2:**
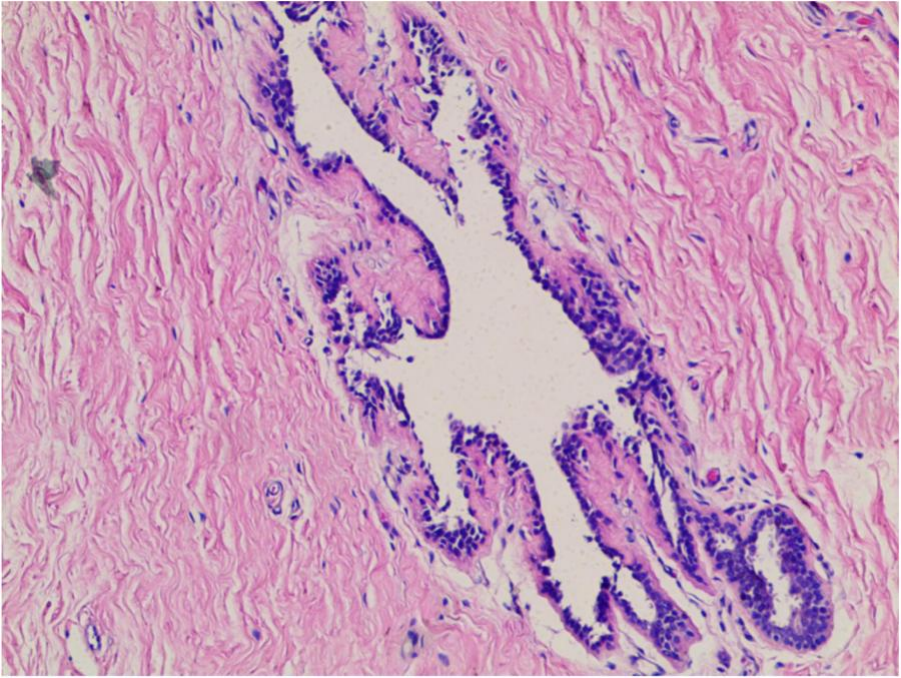
A 24-year-old male patient shown postoperative pathological image.

The operative field was then compressively dressed for ∼8 h to reduce edema and prevent hematoma or seroma formation, and excised specimens were submitted for histopathologic examination (Figure 2).

### 2.3 Postoperative management and follow-up

**Figure 3:**
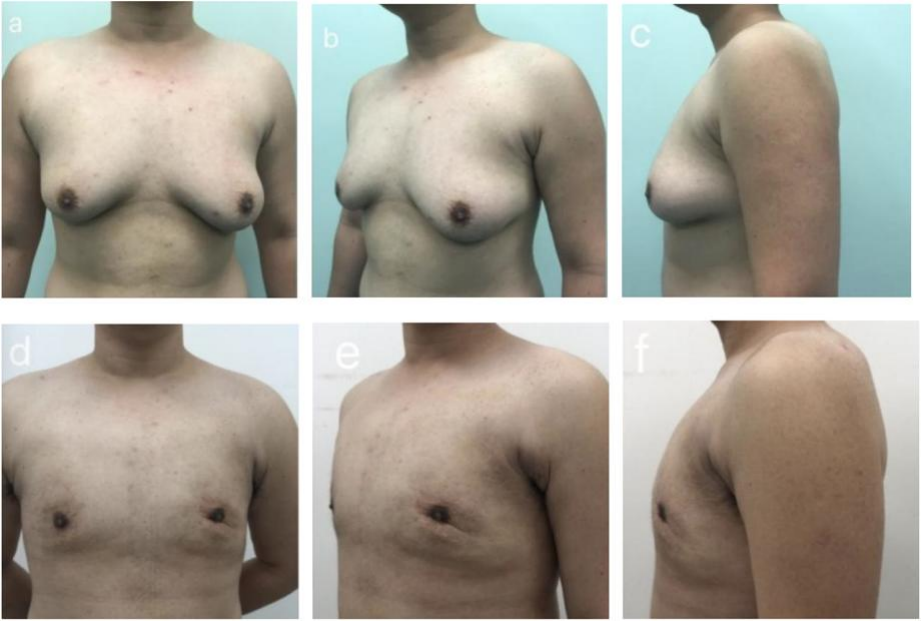
A 19-year-old male patient shown preoperatively at **(a)** front, **(b)** oblique and **(c)** lateral view; postoperative images at 6 months: **(d)** front, **(e)** oblique and **(f)** lateral view.

**Figure 4:**
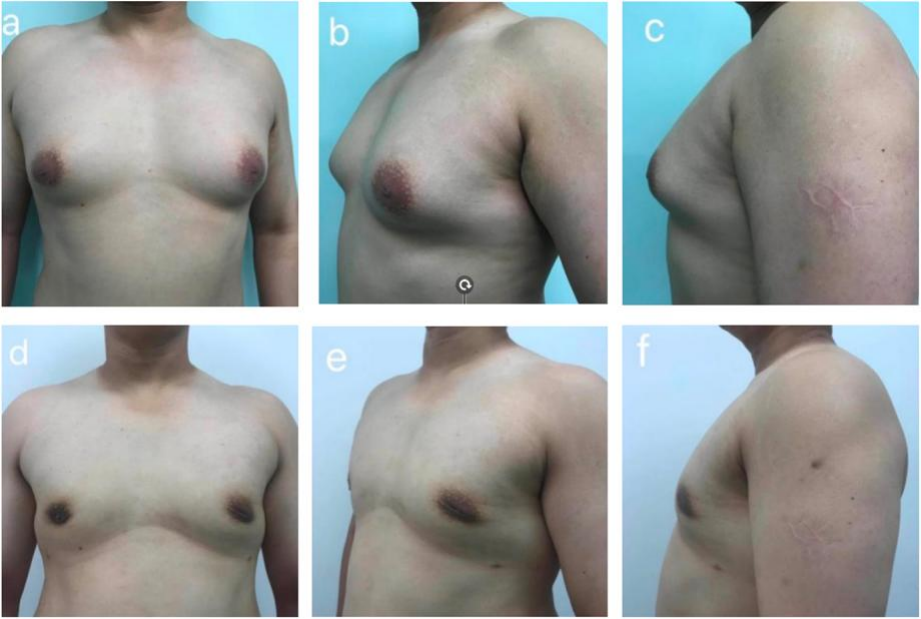
A 28-year-old male patient shown preoperatively at **(a)** front, **(b)** oblique and **(c)** lateral view; postoperative images at 6 months: **(d)** front, **(e)** oblique and **(f)** lateral view.

Compression garments are initiated immediately after surgery and worn continuously for the first month, then during daytime only for the subsequent 8 weeks. Routine follow-up is scheduled at postoperative 3 and 7 days, and at 1 and 6 months. Patient satisfaction was measured using a single-item visual analog scale (VAS) scored from 0 (completely dissatisfied) to 10 (completely satisfied), administered via online follow-up at 6 months postoperatively. This VAS was intended as a measure encompassing satisfaction with chest appearance, scar acceptability, and perceived impact on daily life; it did not separately evaluate bilateral symmetry or nipple–areolar complex (NAC) aesthetics (Figures 3, 4). Standardized clinical photographs were obtained preoperatively and at postoperative follow-up. All images were captured with patients standing in a consistent position with the arms relaxed at the sides, under uniform lighting and at a fixed camera-to-subject distance. Identical views were recorded at each time point. Postoperative photographs were taken at 6 months to minimize the effects of early edema and subcutaneous scar contracture. Patient satisfaction questionnaires were administered online at 1, 3, and 6 months postoperatively, and responses were recorded at each time point.

### 2.4 Statistical analysis

All statistical analyses were performed using SPSS version 26.0 (IBM Corp., Armonk, NY, USA). The distribution of continuous variables was assessed for normality using the Shapiro–Wilk test. Continuous variables with a normal distribution are presented as mean ± standard deviation (SD), whereas non-normally distributed (skewed) continuous variables are reported as median with interquartile range (IQR). Categorical variables are summarized and reported as counts and percentages (*n*, %).

## 3 Results

### 3.1 Patient characteristics

A total of 177 male patients were included in the study. The median age was 28.8 years (range, 11–54) and the median body mass index (BMI) was 27.2 kg/m^2^ (range, 20.9–38.2); 42% were overweight and 24% were obese. Among the cohort, 115 patients had Rohrich grade I–II gynecomastia, while 62 patients had Rohrich grade III–IV disease. Bilateral partial gland excision was performed in 175/177 (99%) patients and unilateral excision in 2/177 (1%); All patients underwent adjunctive liposuction. The median lipoaspirate volume was 882 mL (range, 100–2,500 mL), and the median excised gland weight was 41.9 g (range, 8–180 g). Postoperatively, breast thickness decreased substantially, and the chest contour was smooth and symmetric, with no palpable nodularity or dimpling. The median operative time was 89.7 min (range, 60–120 min). In this study, abnormal hormone values were identified in 71 patients. Elevated estradiol (E2) was observed in 21 patients, decreased total testosterone (T) in 18 patients, and increased testosterone in 6 patients. Notably, 11 patients exhibited concomitant E2 elevation and T reduction. Gonadotropin abnormalities were also detected: follicle-stimulating hormone/luteinizing hormone (FSH/LH) levels were increased in 4 patients and decreased in 5 patients. In addition, elevated prolactin (PRL) was found in 6 patients.

Overall, postoperative complications occurred in 25/177 (14.0%) patients: 19 (10.7%) were mild and 6 (3.3%) were moderate; no major complications were observed. All events were successfully managed within 3 months, with no long-term sequelae. The most common complications were seroma (*n* = 15), hematoma (*n* = 3), surgical-site infection (*n* = 3), and transient cutaneous sensory changes (*n* = 4) (Table 2). Hematomas resolved completely within 2 weeks with aspiration/drainage and localized compression. Infections improved after 3–7 days of oral antibiotics. Sensory symptoms—most commonly mild numbness or reduced nipple sensation—diminished or resolved by the 2-month follow-up, with progressive recovery thereafter. Sensory symptoms—most commonly mild numbness or reduced nipple sensation—diminished or resolved by the 2-month follow-up, with progressive recovery thereafter.

**Table 2:** The characteristics of the patients.

Characteristics	
Median age (range)	28.8 (11–54)
BMI group (range)	27.2 (20.9–38.2)
Normal	34%
Overweight	42%
Obese	24%
Breast surgical	
Unilateral	2 (1%)
Bilateral	175 (99%)
Liposuction volume (mL)	882 (100–2,500)
Total amount of breast removed (g)	41.9 (8–180)
Duration of surgical (min)	89.7 (60–120)
Complication	
Hematoma	15
Seroma	3
Infection	3
Paresthesia	4
No complication	152

BMI, body mass index.

### 3.2 Patient satisfaction (VAS) scores

The median follow-up duration was 8 months. Patient satisfaction was assessed by questionnaire and interview using a visual analog scale (VAS; 0–10). Responses were obtained from all patients at 1 month postoperatively, from 172 patients at 3 months, and from 159 patients at 6 months. The primary analysis was conducted using complete-case data at 6 months, and response rates were reported for each time point. To assess the robustness of findings to missing data, sensitivity analyses using last observation carried forward were performed: for patients missing the 3-month questionnaire, the 1-month assessment was carried forward; for patients missing the 6-month questionnaire, the 3-month assessment was carried forward. The mean satisfaction score was 8.8. Among the evaluated patients, 155 (87.6%) reported being very satisfied, 17 (9.6%) satisfied, and 5 (2.8%) dissatisfied, mainly due to residual adiposity or bilateral asymmetry; of these cases, two were noted at 1 month and three at 2 months postoperatively (Table 3). Overall, patients reported marked improvement in chest appearance, increased self-confidence, and substantially reduced negative impacts on daily activities and social interactions.

**Table 3:** Patient satisfaction (VAS) scores.

Satisfaction	*n* (%)
Very satisfied	155 (87.6)
Satisfied	17 (9.6)
Dissatisfied	5 (2.8)

## 4 Discussion

Peak incidence of gynecomastia occurs across three life stages-neonatal, adolescent, and older adulthood-with adolescence showing the highest rates (9). Although the condition is benign and often self-limiting, resolving spontaneously within 1–3 years in some patients, it can provoke substantial psychological distress and physical discomfort during adolescence. A subset persists beyond 2–3 years, at which point referral for surgical management is commonly considered (3, 10). In this study, most patients underwent surgery between 18 and 35 years, an age group particularly susceptible to peer influence and psychosocial stressors; only three patients were older than 50. Sex-characteristic incongruence of the chest during adolescence can markedly disrupt psychological and social development, leading to interpersonal difficulties, social anxiety, mood disorders (e.g., depression and anxiety), and significant impairment of social and academic functioning. Prior studies demonstrate a positive correlation between gynecomastia severity and psychological distress (10, 11). Surgical intervention is warranted when breast enlargement is conspicuous or when severe psychological distress persists despite conservative medical therapy.

Surgical decision-making should be individualized to each patient's circumstances. Procedures are typically performed in late adolescence (12, 13). The primary goals are to restore a masculine chest contour, efface the inframammary fold, reduce skin laxity, achieve bilateral symmetry, and minimize scarring (14). Although techniques continue to evolve with technological advances, definitive guidelines remain lacking. Earlier literature emphasized direct gland excision, whereas more recent studies favor combining liposuction with gland excision to ensure a smoother postoperative chest. Many prior reports are limited by small sample sizes and thus yield inconclusive results. In this study, we employed liposuction plus minimally invasive periareolar gland excision with follow-up, summarizing the technique's advantages and limitations and analyzing factors associated with surgical outcomes and patient satisfaction.

This procedure entails minimal tissue trauma, facilitates rapid recovery, and is associated with a low complication rate. It leaves no conspicuous scars, typically obviates the need for postoperative drains, and avoids skin excision. Despite its limited incisions, the combined technique enables effective glandular resection while refining chest contour, improving bilateral symmetry, and preserving the native skin envelope, thereby delivering superior aesthetic outcomes. In our experience, it produced favorable cosmetic results in patients with Rohrich grade I–II gynecomastia. However, in more severe cases (Rohrich grade III–IV), liposuction alone or micro-incision excision may be insufficient to achieve satisfactory results and may be associated with postoperative recurrence. In such cases, optimal results often require adjunctive measures—for example, staged or complementary procedures, targeted physical therapy, and, when appropriate, pharmacologic management-to better address skin redundancy, residual glandular/adipose tissue, and patient-specific etiologic factors that may contribute to recurrence. Nevertheless, even among patients with Rohrich grade III–IV disease, postoperative confidence typically improved substantially, particularly in terms of clothed appearance and routine social interactions.

This study used a concealed axillary incision for tumescent infiltration and performed glandular resection through a 0.5-cm periareolar micro-incision at the inferior areolar border. Across the cohort, there was no clinically meaningful increase in incisional pain or conspicuous scar formation; patients reported good comfort, and routine analgesia sufficed without the need for additional medications. The axillary incision, placed within the crease and hair-bearing skin, provided excellent camouflage without affecting hair regrowth, and no hypertrophic scarring was observed on follow-up. The subareolar approach likely preserves the anterior branch of the lateral cutaneous branch of the fourth intercostal nerve that innervates the nipple–areolar complex, facilitating faster sensory recovery while avoiding a semicircular periareolar scar. Although the literature indicates that preserving a 1-cm subareolar disc of glandular tissue can help prevent ischemic necrosis of the nipple–areolar complex (NAC) (15, 16), this may be insufficient in patients with large or highly projected nipples. After tumescent infiltration, NAC edema can further compromise perfusion; in such cases, retaining more than 1 cm of tissue beneath the areola may be necessary to safeguard vascularity and reduce the risk of ischemic necrosis. Overall, the combined axillary–periareolar technique in this cohort balanced aesthetic and functional outcomes, delivering concealed scarring and manageable pain, minimizing tissue trauma, and expediting postoperative recovery.

The overall complication rate in this cohort was 14.0%, consistent with prior reports (3, 16, 17), with hematoma and seroma predominating. Three hematomas occurred within 0–6 h postoperatively: two were managed by evacuation and re-layered compression at the original incision, and one small hematoma resolved with observation and compression alone. Three surgical-site infections were observed: one healed after incision and drainage, and two were controlled with oral antibiotics. Complications clustered in patients with higher BMI (Specifically, two patients had BMI values of 25.4 and 26.4, and the remaining four patients had BMI values greater than 30),implicating obesity-related factors—greater subcutaneous thickness and potential dead space, reduced tissue recoil, and increased local shear—as key contributors. Intraoperatively, overly deep or wide cannula sweeps along the lateral chest wall/anterior axillary region can traction or injure the lateral thoracic vessels and intercostal perforators, promoting early bleeding; this risk is amplified by inadequate tumescent infiltration (insufficient dwell time or uneven distribution) and incomplete punctate hemostasis after broad gland–subdermal undermining, all of which predispose to hematoma/seroma. Postoperatively, insufficient sustained compression, poor garment fit or adherence, and early blood-pressure surges or premature ambulation may precipitate rebleeding. Unaddressed hematoma and sizable dead space provide a favorable medium for bacterial growth-exacerbated by axillary flora, perspiration, and dressing moisture-thereby increasing infection risk. To mitigate these risks, clinical practice should ensure adequate, uniform tumescent infusion with a ≥10–15-minute dwell, maintain superficial-to-mid liposuction planes laterally to limit perforator injury, and perform meticulous point-by-point hemostasis in the gland bed to minimize dead space. Postoperatively, implement continuous, well-fitted, zoned compression for 24–48 h with active adherence monitoring, and schedule early reassessment at 6–12 and 24 h, proceeding to timely evacuation when progressive swelling suggests hematoma. This protocol reduces secondary infection and enhances overall perioperative safety.

The overall satisfaction rate in this cohort was 97.2%, exceeding that reported in prior series (18, 19). Most patients reported increased confidence and were satisfied with scar appearance. Surgical outcomes and satisfaction were influenced by multiple factors, foremost the accuracy of preoperative assessment and the precision of intraoperative technique. A comprehensive preoperative appraisal of the chest—including the sternum, ribs, musculature, adiposity, and skin laxity—guided individualized planning to achieve bilateral symmetry. Asymmetry was documented in two patients at 1 month and three at 2 months, with progressive attenuation between 6 and 12 months. Mechanistically, asymmetry reflects both anatomical and technical contributors: baseline chest-wall incongruities (e.g., differences in bilateral costal-arch and thoracic curvature, sternal rotation, variation in pectoralis thickness/fiber orientation, initial NAC level, and skin elasticity/redundancy) and perioperative factors (e.g., unequal tumescent infiltration or lipoaspirate volumes, disparity in the extent/weight of gland excision, suboptimal compression-garment adherence, and early fibrosis causing localized tethering). Temporally, 1-month asymmetry is more consistent with residual edema, seroma, or early fibrosis and is often reversible, whereas persistent or newly emergent asymmetry at 2 months suggests structural drivers (uneven residual gland/fat or intrinsic chest-wall asymmetry), warranting vigilance for true contour differences rather than transient soft-tissue swelling. For patients with Rohrich grade I–II disease, the postoperative chest contour generally showed no obvious wrinkling or folding. In contrast, patients with Rohrich grade III–IV disease may develop more pronounced wrinkling or folding of the NAC after surgery. Intraoperatively, gentle, meticulous dissection with vascular preservation is essential to minimize tissue injury and reduce hematoma/seroma; postoperatively, adherence to care instructions and scheduled follow-ups supports recovery and maintains results. Psychosocial variables also shape satisfaction: thorough preoperative counseling, patient education, and timely attention to psychological well-being enhance the overall experience. Notably, patients demonstrated high psychological and social satisfaction; psychological satisfaction tended to increase with greater tissue-resection volume, and social satisfaction correlated positively with higher BMI. Although residual fat, glandular tissue, or wrinkling of the NAC can be addressed with secondary procedures, most patients declined further intervention because the feminized chest appearance had been eliminated and a masculine contour restored, leading to regained confidence and resolution of prior inhibitions. As a result, meaningful psychosocial benefits may be realized even when minor residual imperfections remain. Accordingly, high VAS scores may coexist with subtle contour irregularities or visible but acceptable scarring, particularly when the patient's primary preoperative concerns—chest feminization and associated distress—have been substantially relieved.

Despite the outcomes observed in this study—namely a low complication rate and marked improvement in masculine chest contour—several limitations should be acknowledged. First, this was a retrospective study without a control group. Second, no skin excision was performed, which may be suboptimal for patients with substantial skin redundancy. Third, the sample size was relatively limited, which may restrict the generalizability of our findings. In addition, reports of residual adiposity and asymmetry among dissatisfied patients were based on subjective patient feedback, and no postoperative imaging was obtained to objectively quantify these concerns; moreover, patient satisfaction was assessed using a self-developed questionnaire. Finally, the follow-up duration was relatively short, precluding a comprehensive assessment of long-term durability. Future studies should enroll larger cohorts, extend follow-up, adopt validated satisfaction instruments, and ideally employ prospective, controlled (preferably multicenter) designs to more rigorously evaluate the efficacy and safety of this surgical approach.

## 5 Conclusion

This study provides a detailed evaluation of combined liposuction and periareolar micro-incision gland excision for gynecomastia. The approach markedly improved chest appearance and yielded high patient satisfaction across psychological well-being and social interaction domains, while reducing postoperative complications and easing recovery-related pain and functional difficulties. Early postoperative discomfort and asymmetry were generally mild and transient; most symptoms resolved within 1–2 weeks, with asymmetry diminishing progressively on follow-up. By avoiding skin excision, this technique resulted in no conspicuous scarring, further supporting its safety and effectiveness. Although patients with Rohrich grade III–IV gynecomastia may develop postoperative wrinkling or folding of the NAC, the procedure can still improve chest laxity and ptosis and achieve a high level of patient satisfaction. Overall, these findings indicate that liposuction combined with periareolar micro-incision gland excision is an effective treatment for gynecomastia with favorable cosmetic outcomes, particularly in patients with Rohrich grade I–II disease, and may serve as a valuable reference for clinical practice.

## Data Availability

The raw data supporting the conclusions of this article will be made available by the authors, without undue reservation.
